# Low Pathogenicity Avian Influenza (H5N2) Viruses, Dominican Republic

**DOI:** 10.3201/eid2612.200268

**Published:** 2020-12

**Authors:** David H. Chung, Dejelia R. Gomez, Julia M. Vargas, Belkis L. Amador, Mia K. Torchetti, Mary L. Killian, David E. Swayne, Dong-Hun Lee

**Affiliations:** University of Connecticut, Storrs, Connecticut, USA (D.H. Chung, D.-H. Lee);; Ministry of Agriculture, Roseau, Dominican Republic (D.R. Gomez, J.M. Vargas, B.L. Amador);; US Department of Agriculture, Athens, Georgia, USA (D.E. Swayne);; US Department of Agriculture, Ames, Iowa, USA (M.K. Torchetti, M.L. Killian)

**Keywords:** epidemiology, low pathogenicity avian influenza virus, H5N2, wild bird, poultry, phylogenetic analysis, viruses, influenza, respiratory infections, zoonoses, vector-borne infections, Dominican Republic

## Abstract

Low pathogenicity avian influenza (H5N2) virus was detected in poultry in the Dominican Republic in 2007 and re-emerged in 2017. Whole-genome sequencing and phylogenetic analysis show introduction of an H5N2 virus lineage from Mexico into poultry in the Dominican Republic, then divergence into 3 distinct genetic subgroups during 2007–2019.

Low pathogenicity avian influenza virus (LPAIV) subtype H5N2 has caused outbreaks in poultry in Mexico since 1993 and mutated into highly pathogenic avian influenza virus (HPAIV) H5N2 during 1994–1995 (*1*). In 1994, a vaccination program against H5N2 in poultry was established in Mexico; HPAIV H5N2 was eradicated there in 1995 (*2*). However, LPAIV H5N2 persisted and related viruses spread to neighboring countries (*1*,*3*,*4*). In addition, the H5N2 virus lineage from Mexico was introduced to Taiwan in 2003, likely because of inadequately inactivated vaccines. The virus then reassorted with the local avian influenza (H6N1) virus strain that has been enzootic in chickens in Taiwan since 1997 to produce reassortant H5N2 virus possessing hemagglutinin (HA) and neuraminidase (NA) genes of H5N2 virus from Mexico and internal genes of the Taiwan H6N1 virus. The reassortant H5N2 virus mutated into an HPAIV and caused outbreaks in poultry in Taiwan during 2012 (*5*).

In 2007, outbreaks of LPAIV H5N2 occurred among chickens in Santo Domingo and Higüey-La Otra Banda, Dominican Republic, and were reported to the World Organisation for Animal Health (OIE) (*6*). The OIE Reference Laboratory detected LPAIV H5N2 lineages from Mexico in samples from the Dominican Republic outbreaks on December 21, 2007. During December 2007–February 2008, a total of 11 avian influenza A viruses were detected in the Dominican Republic from backyard birds, fighting birds, and a live bird market (*7*) (Appendix Table 1). During 2007–2016, serologic surveillance of poultry detected 364/45,440 (0.80%) samples exhibiting positive antibody responses, suggesting low level circulation of LPAIVs in the Dominican Republic, but the HA and NA subtypes were not identified (data not shown). During September–November 2017, the H5N2 LPAIV re-emerged and affected 5 commercial chicken farms in Espaillat, San Juan, and La Vega (Appendix Table 2). Subsequently, the viruses were detected in 23 commercial and backyard poultry flocks during September 2018–February 2019 (*8*). During the 2017–2019 H5N2 LPAIV outbreak period, seropositivity reached 52% (8,740/16,543).

Since 2007, limited information on H5N2 LPAIVs and few genetic sequences have been reported. We provide sequenced genomes of 19 H5N2 LPAIVs identified in the Dominican Republic during 2007–2019: 1 virus from 2007, 6 from 2017, 1 from 2018, and 11 from 2019.

The 2007 H5N2 LPAIV, Ck/Dominican_Republic/2007(H5N2), had an HA cleavage site sequence with 2 basic amino acids (PQRETR/G). However, the 2017–2019 viruses possessed 3 monobasic amino acids (PQRGKR/G, PQREKR/G, and LQREKR/G) (Appendix Table 3). Seven representative isolates were of low pathogenicity in chickens on intravenous inoculation (intravenous pathogenicity index = 0.0). The acquisition of an additional basic amino acid in the HA cleavage site raises a concern regarding the increased risk for mutation to an HPAIV.

All genes formed a well-supported monophyletic clade (bootstrap support of 98–100 in maximum likelihood phylogeny and posterior probability of 0.99–1.00 in Bayesian phylogeny), suggesting their close relationship from a single viral introduction into the poultry population descended from A/Ck/Hidalgo/28159–232/94 (H5N2)–like virus and maintenance in poultry in the Dominican Republic (Figure 1; Appendix Figures 1–8). The inferred time to most recent common ancestor (tMRCA) for each gene of H5N2 viruses identified in the Dominican Republic ranged from February 2005 to August 2006, suggesting that ancestors of these viruses emerged from an H5N2 virus lineage introduced from Mexico during this period (Appendix Table 4). Phylogenetic analyses show divergence of all 8 gene segments into 3 genetic sublineages, designated as sublineage A, which contains viruses collected in 2017 and 2019; B1, which contains viruses collected during 2017; and B2, which contains viruses collected during 2018–2019. The 8 gene segments of Ck/Dominican_Republic/044726_013/2018(H5N2) fell into sublineage B (Appendix Table 3).

The prediction of N-linked glycosylation sites in the HA protein revealed that the H5N2 LPAIVs have 9 potential glycosylation sites at position 27, 39, 142, 181, 252, 277, 302, 496, and 555 (H5 numbering system), with a range of 6–8 sites for individual isolates (Appendix Table 5, Figure 9). The potential glycosylation at positions 142, 181, and 252 were found within antigenic sites (*9*). The initially identified Ck/Dominican_Republic/2007(H5N2) was predicted to contain N-linked glycosylation at the antigenic sites at positions 181 and 252. Sublineage A and B1 did not have the predicted glycosylation at position 252 but gained additional glycosylation at position 142. However, sublineage B2 was predicted to possess glycosylation at all antigenic sites at positions 142, 181, and 252, except for the Ck/Dominican_Republic/020695-012-1107-19-16/2019 (H5N2) strain, which has N-glycosylation only at positions 142 and 252. 

Enhanced active surveillance is required to monitor the evolution and spread of H5N2 viruses in the Dominican Republic; such efforts could further the epidemiologic understanding and the design of improved prevention strategies. Additional studies could elucidate whether the genetic changes in glycosylation and antigenic sites contribute to the alterations in antigenicity of the H5N2 LPAIV from the Dominican Republic against current H5 virus vaccine strains. 

**Figure:**
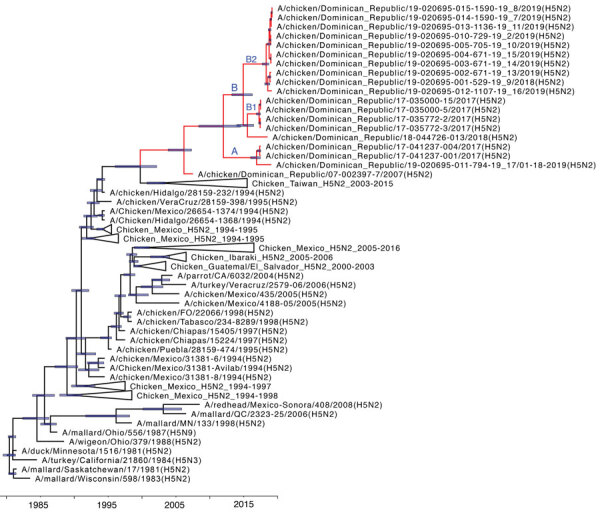
Relaxed clock molecular phylogenetic tree for hemagglutinin gene of low pathogenicity avian influenza (H5N2) viruses, Dominican Republic. The phylogenetic relationships and temporal evolutionary history have been estimated by molecular clock analysis. Red text indicates the monophyletic Dominican Republic influenza H5N2 viruses. Node bars indicate 95% credibility intervals on node ages. Scale bar shows time in years.

Appendix Additional information on materials and methods used to sequence and determine phylogeny of low pathogenicity avian influenza (H5N2) viruses in the Dominican Republic.
