# Supplementary material for: Low Pathogenicity Avian Influenza (H5N2) Viruses, Dominican Republic
Source: Emerg Infect Dis. 2020 Dec;26(12):3094–6. doi: 10.3201/eid2612.200268 (PMC7706952; doi:10.3201/eid2612.200268)
Supplement: Appendix — Additional information on materials and methods used to sequence and determine phylogeny of low pathogenicity avian influenza (H5N2) viruses in the Dominican Republic. [file 20-0268-Techapp-s1.pdf]

Article DOI: https://doi.org/10.3201/eid2612.200268

Mexican-Lineage Low Pathogenicity Avian Influenza (H5N2) Viruses, Dominican Republic

Appendix

Materials and Methods

We sequenced complete genomes of 19 low pathogenicity avian influenza viruses (LPAIVs) identified in the Dominican Republic during 2007–2019, including 1 from 2007, 6 from 2017, 1 from 2018, and 11 from 2019. We extracted viral RNA by using the MagMAX Viral RNA Isolation Kit (Ambion/ThermoFisher Scientific, https://www.thermofisher.com). All 8 segments of isolates were amplified by multi-segment reverse transcription-PCR (*1*) and whole-genome sequencing was conducted by using the Miseq system (Illumina, https://www.illumina.com). We used the Nextera XT DNA Sample Preparation Kit (Illumina) to generate multiplexed paired-end sequencing libraries, according to the manufacturer's instructions. The dsDNA was fragmented and tagged with adapters by Nextera XT transposase and 12-cycle PCR amplification. Fragments were purified on Agencourt AMpure XP beads (Beckman Coulter, https://www.beckmancoulter.com) and analyzed on a High Sensitivity DNA Chip on the Bioanalyzer (Agilent Technologies, https://www.agilent.com). The barcoded multiplexed library sequencing was performed by using the 250 cycle MiSeq Reagent Kit v2 (Illumina). We performed de novo and directed assembly of genome sequences by using SeqMan NGen version 4 (https://www.nihlibrary.nih.gov/resources/tools/seqman-ngen). We deposited nucleotide sequences in GenBank (Appendix Table 3).

For phylogenetic analysis, we downloaded all sequences of H5N2 virus lineages from Mexico were downloaded from the National Center for Biotechnology Information Influenza Virus Resource database (https://www.ncbi.nlm.nih.gov) and added to sequence alignments in September 2019. On the basis of BLAST (http://blast.ncbi.nlm.nih.gov/Blast.cgi) searches of HA and NA genes, North American lineage avian influenza virus sequences that were collected

before the emergence of H5N2 lineage from Mexico were added. Multiple sequence alignments were prepared using Multiple Alignment with Fast Fourier Transformation (MAFFT; https://mafft.cbrc.jp), and manual optimization of the alignment was done by using BioEdit (https://bioedit.org) to trim nucleotide positions to only use protein coding regions for phylogenetic analyses using 70 sequences each for polymerase (PA), polymerase basic 2 [PB2] , PB1, and matrix (M) segments; 71 sequences for nonstructural protein (NSP); 73 sequences for neuraminidase (NA) segment; 180 hemagglutinin (HA) segment; and 68 sequences for nucleoprotein (NP) segment. Maximum-likelihood (ML) phylogenies of each gene segment were estimated with RAxML program (*2*) using the GTR model of nucleotide substitution with gamma-distributed site heterogeneity rate variation. Bootstrap analysis was conducted via rapid bootstrap with 1,000 replicates for statistical support of generated tree topology.

The Bayesian relaxed clock phylogenetic analysis of the 8 segments was performed by using BEAST version 1.10.4 (*3*). TempEst software (https://www.beast.community/tempest) was used to investigate suitable correlation of the temporal signal and clock-likeness of phylogenies for phylogenetic molecular clock BEAST analysis based on collection date and genetic divergence (*4*). We applied an uncorrelated lognormal distribution relaxed clock method, the Hasegawa-Kishino-Yano (HKY) nucleotide substitution model with 4 categories and the Gaussian Markov random field (GMRF) Bayesian skyride coalescent prior (*5*). We ran a Markov chain Monte Carlo (MCMC) method to sample trees and ran evolutionary parameters for 5.0×10^7 generations for HA gene and 3.0×10^7 generations for the other 7 genes. We combined $\geq$3 independent chains to ensure adequate sampling of the posterior distribution of trees. We used TRACER version 1.7.1 (http://beast.community/tracer) with 10% burn-in to confirm convergence of each BEAST output and sufficient effective sample size (>200). We generated a maximum clade credibility (MCC) tree for each dataset by using TreeAnnotator in BEAST. We used FigTree 1.4.4 (http://tree.bio.ed.ac.uk) for visualization of MCC trees and estimated the time to the most recent common ancestor (tMRCA) of each clade.

We predicted potential N-glycosylation sites by using NetNGlyc server 1.0 (http://www.cbs.dtu.dk/services/NetNGlyc) and GlyProt (http://www.glycosciences.de/modeling). We modeled the HA structure by using the previously published 3-dimensional (3D) structure (PDB ID: 5ykc) in the SWISS-MODEL server (https://swissmodel.expasy.org). We created visualizations of 3D HA structures by using the

PyMOL Molecular Graphics System Version 2.0 (Schrödinger, LLC, https://www.schrodinger.com).

For the intravenous pathogenicity index of viruses, 10 6-week-old specific pathogen free chickens were inoculated intravenously with 0.1 ml of 1:10 dilutions of infectious allantoic fluids. The intravenous pathogenicity index (IVPI) was calculated according to the World Organization for Animal Health standard protocol (http://www.oie.int/international-standard-setting/terrestrial-code). Isolates with an IVPI >1.2 were determined to be HPAI. The challenge study and all experiments with live viruses were conducted in a Biosafety Level 3 facility.

References

1. Chrzastek K, Lee DH, Smith D, Sharma P, Suarez DL, Pantin-Jackwood M, et al. Use of Sequence-Independent, Single-Primer-Amplification (SISPA) for rapid detection, identification, and characterization of avian RNA viruses. Virology. 2017;509:159–66. PubMed https://doi.org/10.1016/j.virol.2017.06.019

2. Stamatakis A. RAxML version 8: a tool for phylogenetic analysis and post-analysis of large phylogenies. Bioinformatics. 2014;30:1312–3. PubMed https://doi.org/10.1093/bioinformatics/btu033

3. Drummond AJ, Rambaut A. BEAST: Bayesian evolutionary analysis by sampling trees. BMC Evol Biol. 2007;7:214. PubMed https://doi.org/10.1186/1471-2148-7-214

4. Rambaut A, Lam TT, Max Carvalho L, Pybus OG. Exploring the temporal structure of heterochronous sequences using TempEst (formerly Path-O-Gen). Virus Evol. 2016;2:vew007. PubMed https://doi.org/10.1093/ve/vew007

5. Minin VN, Bloomquist EW, Suchard MA. Smooth skyride through a rough skyline: Bayesian coalescent-based inference of population dynamics. Mol Biol Evol. 2008;25:1459–71. PubMed https://doi.org/10.1093/molbev/msn090

Appendix Table 1. Summary of World Organization for Animal Health report on Mexican-lineage low pathogenicity avian influenza H5N2 virus outbreak, Dominican Republic, December 2017–February 2018

No.	Date	Region	Unit	No. susceptible	No. cases	No. deaths	No. killed and disposed	No. slaughtered	Affected population
1	2007 Dec 12	Higuey, La Otra Banda, La Altagracia	Village	15	1	0	0	15	–
2	2007 Dec 12	Traspatio, Santo Domingo, Distrito Nacional	Village	115	1	0	0	115	Live bird market
3	2007 Dec 21	San Pedro de Macoris, San Pedro de Macoris, San Pedro De Macoris	Village	5	1	0	5	0	–
4	2008 Jan 4	La Vega, Bacuí, Barranca, La Vega	Village	26	1	0	26	0	Fighting cock from Higuey
5	2008 Jan 10	Villa El Sombrero, El Sombrero, Baní, Peravia	Village	114	1	0	114	0	–
6	2008 Jan 11	La Otra Banda, Cruce del Isleño, La Otra Banda, La Altagracia	Village	330	0	0	0	330	Fighting birds
7	2008 Jan 16	Santo Domingo Oeste, Hato Nuevo, Santo Domingo, Distrito Nacional	Village	205	1	0	205	–	Fighting cocks
8	2008 Jan 18	Batey Central, Barahona	Village	30	0	0	0	30	Fighting cocks
9	2008 Jan 26	Corbano Sur, San Juan	Village	5	0	0	0	5	Backyard birds
10	2008 Jan 28	Villa Gonzalez, La Delgada, Villa Gonzalez, Santiago	Village	22	0	0	0	22	Fighting cocks
11	2008 Feb 4	Pedernales, Pedernales	Village	4	0	0	0	4	Fighting cocks
Total	–	–	–	871	6	0	350	521	–

Statistics	Apparent morbidity rate, %	Apparent mortality rate, %	Apparent case fatality rate, %	Proportion susceptible animals lost, %
	0.69	0.00	0.00	100.00

Appendix Table 2. Summary of World Organization for Animal Health report on Mexican-lineage low pathogenicity avian influenza H5N2 virus outbreaks, Dominican Republic, September 2017–June 2019

No.	Date	Region	Epidemiology Unit	No. susceptible	No. cases	No. deaths	No. killed and disposed	No. slaughtered	Affected population
12	2017 Sep 30	Espaillat, Guanabano, Moca, Espaillat	Farm	48,877	1,953	1,953	46,924	0	Population kept in a farm composed of 8 layer units
13	2017 Oct 10	San Juan, Sabana Alta, San Juan de la Maguana, San Juan	Farm	46,500	12,657	12,657	33,843	0	Broiler chickens in 3 sheds
14	2017 Oct 10	La Vega, Río Verde Arriba, Arroyo Hondo, Rio Verde Arriba, La Vega	Farm	34,000	1,639	1,639	32,361	0	Layer hens in 5 sheds
15	2017 Oct 30	La Vega, Cutupú, Río Verde Arriba, La Vega	Farm	27,410	1,257	1,257	26,153	0	Layer hens in 4 sheds
16	2017 Dec 11	La Vega, Cutupú, Cutupú, Rio Verde Arriba, La Vega	Farm	35,000	43	43	34,957	0	Layer hens in production
17	2018 Jan 9	Salcedo/Hermanas Mirabal, Jayabo, Salcedo, Salcedo	Farm	11,500	785	785	10,715	0	Layer hens in 2 sheds
18	2018 Jan 9	Salcedo/Hermanas Mirabal, Jayabo, Salcedo, Salcedo	Farm	32,400	–	0	32,400	0	Layer hens in production
19	2018 Jan 10	La Vega, Rio Verde Arriba, Arroyo Hondo, Rio Verde Arriba, La Vega	Farm	26	6	0	26	0	Backyard hens
20	2018 Mar 20	La Vega, Río Verde Arriba, Cutupú, Rio Verde Arriba, La Vega	Farm	34,500	30	30	34,470	0	Layer hens distributed between 3 coops
21	2018 Apr 2	La Vega, Caguey, Rio Verde Arriba, La Vega	Farm	22,965	46	46	22,919	0	Layer hens distributed between 4 coops
22	2018 Nov 2	Puerto Plata, Cayacoa, Luperón, Puerto Plata	Backyard	1,126	773	745	136	217	Backyard birds (hens, ducks, turkeys and guinea fowls)
23	2018 Dec 30	Espaillat, El Corozo, Moca, Espaillat	Farm	1,800	30	–	0	0	Family farm with Israeli chicks
24	2018 Dec 27	Puerto Plata, Cayacoa, Luperón, Puerto Plata	Backyard	55	7	7	48	0	Laying hens
25	2019 Jan 8	Espaillat, Quebrada Honda, Moca, Espaillat	Backyard	56,685	2,350	2,350	0	54,335	Family farm with heavy birds
26	2019 Jan 8	Espaillat, El Corozo, Moca, Espaillat	Farm	3,000	27	–	0	0	Family farm with Israeli chicks
27	2019 Jan 10	Espaillat, El Caminito de la Rosario, Moca, Espaillat	Farm	45,000	535	530	0	0	Light layer birds
28	2019 Jan 14	Espaillat, Hincha, Moca, Espaillat	Farm	18,000	10	–	0	17,990	Light layer birds
29	2019 Jan 21	Espaillat, Las Lagunas, Moca, Espaillat	Farm	59,097	21	–	0	0	Backyard poultry
30	2019 Jan 22	Espaillat, Monte de la Jagua, Moca, Espaillat	Backyard	19	12	7	0	0	Backyard poultry

No.	Date	Region	Epidemiology Unit	No. susceptible	No. cases	No. deaths	No. killed and disposed	No. slaughtered	Affected population
31	2019 Jan 24	Santo Domingo, Mal Nombre, La Victoria, Distrito Nacional	Backyard	50	30	–	0	0	Fighting cocks
32	2019 Feb 5	Espaillat, Juan López, Moca, Espaillat	Farm	14,170	2,330	2,330	0	10,050	Broilers
33	2019 Feb 5	Espaillat, Cuero Duro, San Victor, Espaillat	Farm	3,500	550	550	0	2,950	Broilers
34	2019 Feb 10	Salcedo, La ceiba, Villa Tapa, Salcedo	Farm	96,963	9	6	0	0	Light layers
35	2019 Feb 21	La Vega, Monte Adentro, La Vega, La Vega	Farm	3,500	10	5	0	3,490	Light layers
36	2019 Feb 23	San José de Ocoa, El Estrecho, Rancho Arriba, San Jose De Ocoa	Farm	45,000	7,145	2,140	37,855	0	Heavy layers
37	2019 May 8	Santiago, Tamboril, Tamboril, Santiago	Farm	247,718	5	–	0	247,713	Broilers
38	2019 May 22	La Vega, Rio Verde, La Vega, La Vega	Farm	19,500	1	–	0	0	Light layers
39	2019 Jun 2	Espaillat, Las Barias, Moca, Espaillat	Farm	25,000	11	–	0	24,989	Israeli-bred hens
Total	–	–	–	933,361	32,272	27,080	312,807	361,734	–

Statistics	Apparent morbidity rate, %	Apparent mortality rate, %	Apparent case fatality rate, %	Proportion susceptible animals lost, %
	3.46	2.90	83.91	75.17

Appendix Table 3. Strain, collection date, and GenBank accession numbers for low pathogenicity avian influenza H5N2 viruses used in this study

Strain	Collection date	Accession no.	Segments (sublineages)								HA cleavage site motif	IVPI
			PB2	PB1	PA	HA	NP	NA	M	NS		
A/CK/DR/07_002397_7/2007	2007	MN882390–7	–	–	–	–	–	–	–	–	PQRETR/G	0
A/CK/DR/17_041237_001/2017	2017	MN882430–37	A	A	A	A	A	A	A	A	PQRGKR/G	0
A/CK/DR/17_035772_2/2017	2017	MN882414–21	B1	B1	B1	B1	B1	B1	B1	B1	PQREKR/G	ND
A/CK/DR/17_035772_3/2017	2017	MN882422–29	B1	B1	B1	B1	B1	B1	B1	B1	PQREKR/G	ND
A/CK/DR/17_035000_5/2017	2017	MN882398–405	B1	B1	B1	B1	B1	B1	B1	B1	PQREKR/G	0
A/CK/DR/17_035000_15/2017	2017	MN882406–13	B1	B1	B1	B1	B1	B1	B1	B1	PQREKR/G	0
A/CK/DR/17_041237_004/2017	2017	MN882438–45	A	A	A	A	A	A	A	A	PQRGKR/G	ND
A/CK/DR/18_044726_013/2018	2018	MN882446–53	B	B	B	B	B	B	B	B	PQREKR/G	0
A/CK/DR/18_020695_001_529_19_9/2018	2018 Dec 13	MN882454–61	B2	B2	B2	B2	B2	B2	B2	B2	LQREKR/G	0
A/CK/DR/19_020695_002_671_19_13/2019	2019 Jan 9	MN882462–69	B2	B2	B2	B2	B2	B2	B2	B2	LQREKR/G	ND
A/CK/DR/19_020695_003_671_19_14/2019	2019 Jan 9	MN882470–77	B2	B2	B2	B2	B2	B2	B2	B2	LQREKR/G	ND
A/CK/DR/19_020695_004_671_19_15/2019	2019 Jan 9	MN882478–85	B2	B2	B2	B2	B2	B2	B2	B2	LQREKR/G	ND
A/CK/DR/19_020695_005_705_19_10/2019	2019 Jan 11	MN882486–93	B2	B2	B2	B2	B2	B2	B2	B2	LQREKR/G	ND
A/CK/DR/19_020695_010_729_19_2/2019	2019 Jan 15	MN882494–501	B2	B2	B2	B2	B2	B2	B2	B2	LQREKR/G	ND
A/CK/DR/19_020695_011_794_19_17/2019	2019 Jan 18	MN882502–509	A	A	A	A	A	A	A	A	LQREKR/G	0
A/CK/DR/19_020695_012_1107_19_16/2019	2019 Feb 18	MN882510–517	B2	B2	B2	B2	B2	B2	B2	B2	PQRGKR/G	ND
A/CK/DR/19_020695_013_1136_19_11/2019	2019 Feb 18	MN882518–525	B2	B2	B2	B2	B2	B2	B2	B2	LQREKR/G	ND
A/CK/DR/19_020695_014_1590_19_7/2019	2019 Mar 25	MN882526–533	B2	B2	B2	B2	B2	B2	B2	B2	LQREKR/G	ND
A/CK/DR/19_020695_015_1590_19_8/2019	2019 Mar 25	MN882534–541	B2	B2	B2	B2	B2	B2	B2	B2	LQREKR/G	ND

*CK, chicken, DR, Dominican Republic; IVPI, intravenous pathogenicity index; ND, not done; –, initially detected H5N2 virus in Dominican Republic.

Appendix Table 4. Estimated time to the most recent common ancestor (tMRCA) for each gene segment isolated with 95% highest posterior density, Dominican Republic

Segment	tMRCA	95% HPD interval		Posterior
		Begin	End	
PB2	2006 Aug 29	2004 Jan 3	2007 Jul 2	1
PB1	2006 Aug 6	2002 Jan 18	2007 Jul 2	0.99
PA	2005 Jun 22	2002 Mar 12	2007 Apr 16	1
HA	2006 Mar 8	2003 Nov 8	2007 Apr 17	0.99
NP	2005 Feb 27	2001 May 8	2007 May 11	1
NA	2006 Mar 4	2003 Aug 8	2007 Jun 7	1
M	2006 Mar 9	2003 May 19	2007 Jun 2	1
NS	2005 May 1	2001 Jan 5	2007 Mar 6	1

*tMRCA were calculated by using bayesian evolutionary analysis sampling trees (BEAST). HPD, highest posterior density; PB2, polymerase basic 2; PB1, polymerase basic 1; PA, polymerase acidic; HA, hemagglutinin; NP, Nucleoprotein; NA, neuraminidase; M, matrix protein; NS, nonstructural protein, tMRCA, inferred most recent common ancestor.

Appendix Table 5. Predicted N-glycosylation sites in HA proteins of avian influenza H5N2/Dominican Republic Strains

	Glycosylation site (H5 numbering)								
Strain/genetic cluster	27	39	142*	181*	252*	277	302	496	555
A/chicken/Dominican_Republic/07_002397_7/2007(H5N2)	NSTK	NVTV		NNTN	NDSI		NSSM	NGTY	NGSL
A/chicken/Dominican_Republic/17_041237_001/2017(H5N2) / A	NSTT	NVTV	NASA	NNTN			NSSM	NGTY	NGSL
A/chicken/Dominican_Republic/17_041237_004/2017(H5N2) / A	NSTT	NVTV	NASA	NNTN			NSSM	NGTY	NGSL
A/chicken/Dominican_Republic/19_020695_011_794_19_17/2019(H5N2) / A	NSTT	NVTV	NASA	NNTN			NSSM	NGTY	NGSL
A/chicken/Dominican_Republic /18_044726_013/2018(H5N2) / B	NSTK	NVTV	NASA	NNTN			NSSL	NGTY	NGSL
A/chicken/Dominican_Republic/17_035772_2/2017(H5N2) / B1	NSTK	NVTV	NATA	NNTN			NSSL	NGTY	
A/chicken/Dominican_Republic/17_035000_5/2017(H5N2) / B1	NSTK	NVTV	NATA	NNTN			NSSL	NGTY	
A/chicken/Dominican_Republic/17_035000_15/2017(H5N2) / B1	NSTK	NVTV	NATA	NNTN			NSSL	NGTY	
A/chicken/Dominican_Republic /17_035772_3/2017(H5N2) / B1	NSTK	NVTV	NATA	NNTN		NSTI	NSSL	NGTY	
A/chicken/Dominican_Republic /19_020695_010_729_19_2/2019(H5N2) / B2	NSTT	NVTV	NASA	NNTN	NDSI		NSSL	NGTY	NGSL
A/chicken/Dominican_Republic /19_020695_014_1590_19_7/2019(H5N2) / B2	NSTT	NVTV	NASA	NNTN	NDSI		NSSL	NGTY	NGSL
A/chicken/Dominican_Republic /19_020695_015_1590_19_8/2019(H5N2) / B2	NSTT	NVTV	NASA	NNTN	NDSI		NSSL	NGTY	NGSL
A/chicken/Dominican_Republic /19_020695_001_529_19_9/2019(H5N2) / B2	NSTT	NVTV	NASA	NNTD	NDSI		NSSL	NGTY	NGSL
A/chicken/Dominican_Republic /19_020695_005_705_19_10/2019(H5N2) / B2	NSTT	NVTV	NASA	NNTN	NDSI		NSSL	NGTY	NGSL
A/chicken/Dominican_Republic /19_020695_013_1136_19_11/2019(H5N2) / B2	NSTT	NVTV	NASA	NNTN	NDSI		NSSL	NGTY	NGSL
A/chicken/Dominican_Republic /19_020695_002_671_19_13/2019(H5N2) / B2	NSTT	NVTV	NASA	NNTD	NDSI		NSSL	NGTY	NGSL
A/chicken/Dominican_Republic /19_020695_003_671_19_14/2019(H5N2) / B2	NSTT	NVTV	NASA	NNTN	NDSI		NSSL	NGTY	NGSL
A/chicken/Dominican_Republic /19_020695_004_671_19_15/2019(H5N2) / B2	NSTT	NVTV	NASA	NNTN	NDSI		NSSL	NGTY	NGSL
A/chicken/Dominican_Republic /19_020695_012_1107_19_16/2019(H5N2) / B2	NSTT	NVTV	NASA		NDSI		NSSL	NGTY	NGSL

*Predicted N-glycosylation sites on antigenic site.

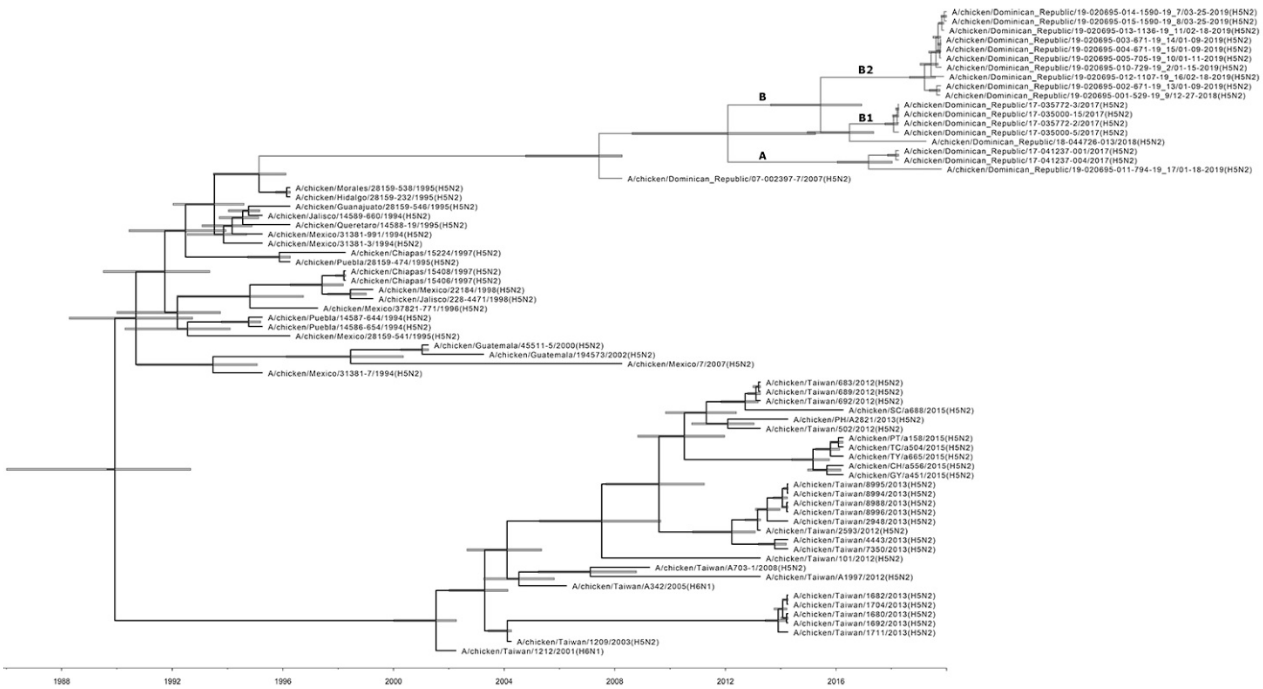

Appendix Figure 1. Maximum-likelihood phylogeny for the PB2 genome segment sequences of low pathogenicity avian influenza H5N2 viruses, Dominican Republic. Bootstrap values >70% are shown. The scale bar indicates the number of nucleotide substitutions per site.

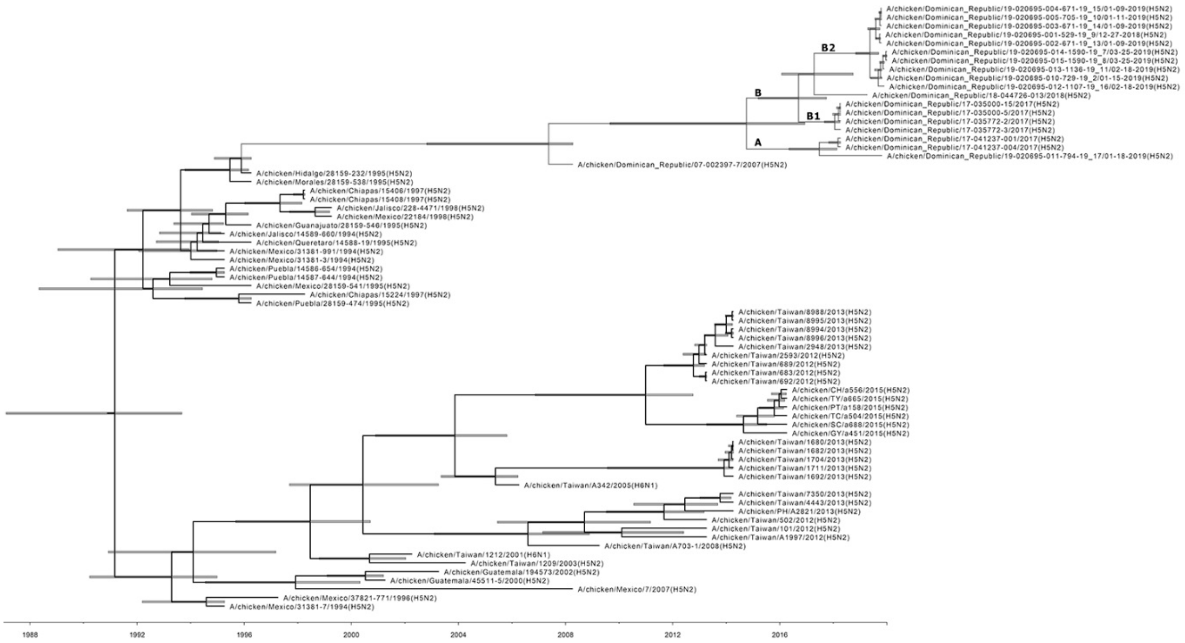

Appendix Figure 2. Maximum-likelihood phylogeny for the PB1 genome segment sequences of low pathogenicity avian influenza H5N2 viruses, Dominican Republic. Bootstrap values >70% are shown. The scale bar indicates the number of nucleotide substitutions per site.

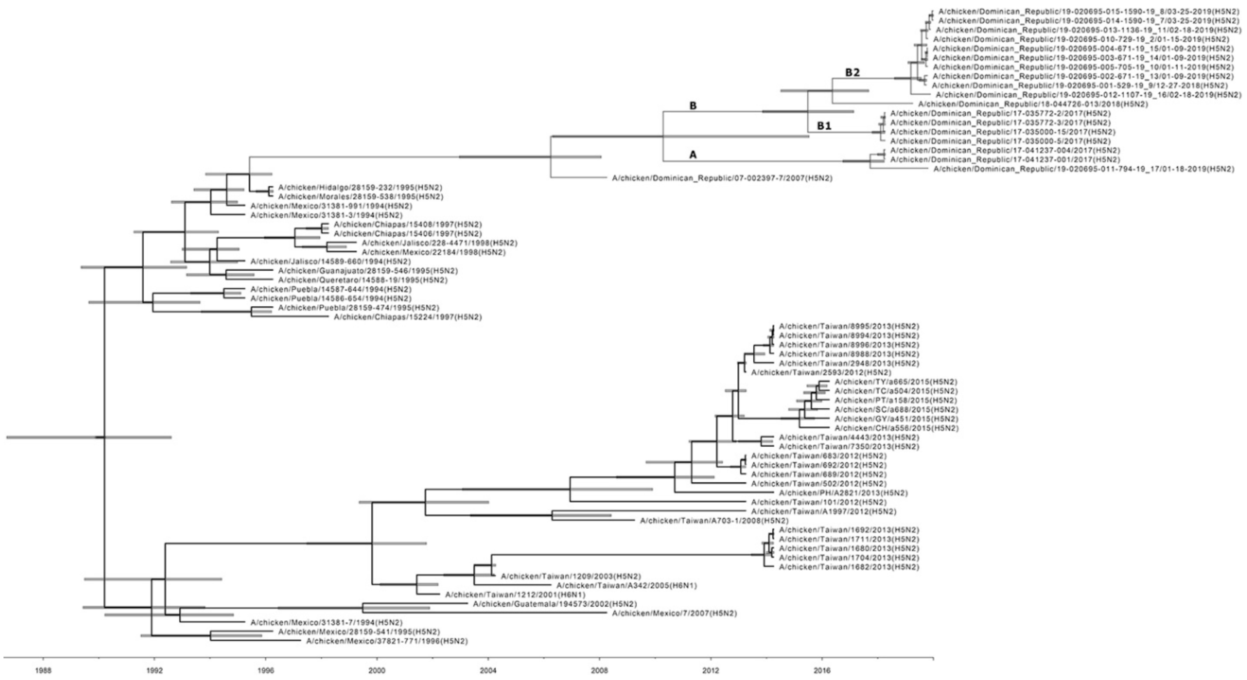

Appendix Figure 3. Maximum-likelihood phylogeny for the PA genome segment sequences of low pathogenicity avian influenza H5N2 viruses, Dominican Republic. Bootstrap values >70% are shown. The scale bar indicates the number of nucleotide substitutions per site.

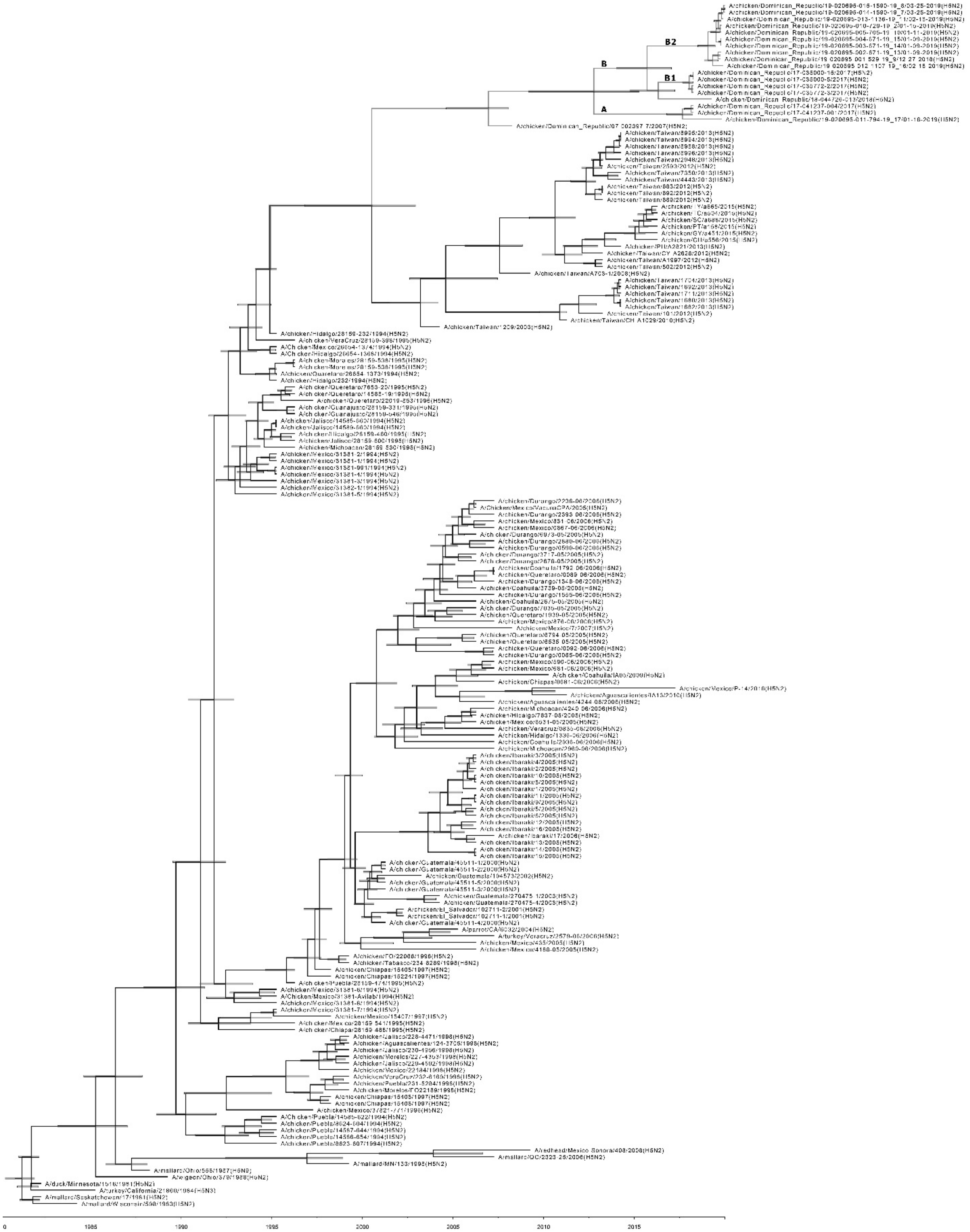

Appendix Figure 4. Maximum-likelihood phylogeny for the HA genome segment sequences of low pathogenicity avian influenza H5N2 viruses, Dominican Republic. Bootstrap values >70% are shown. The scale bar indicates the number of nucleotide substitutions per site.

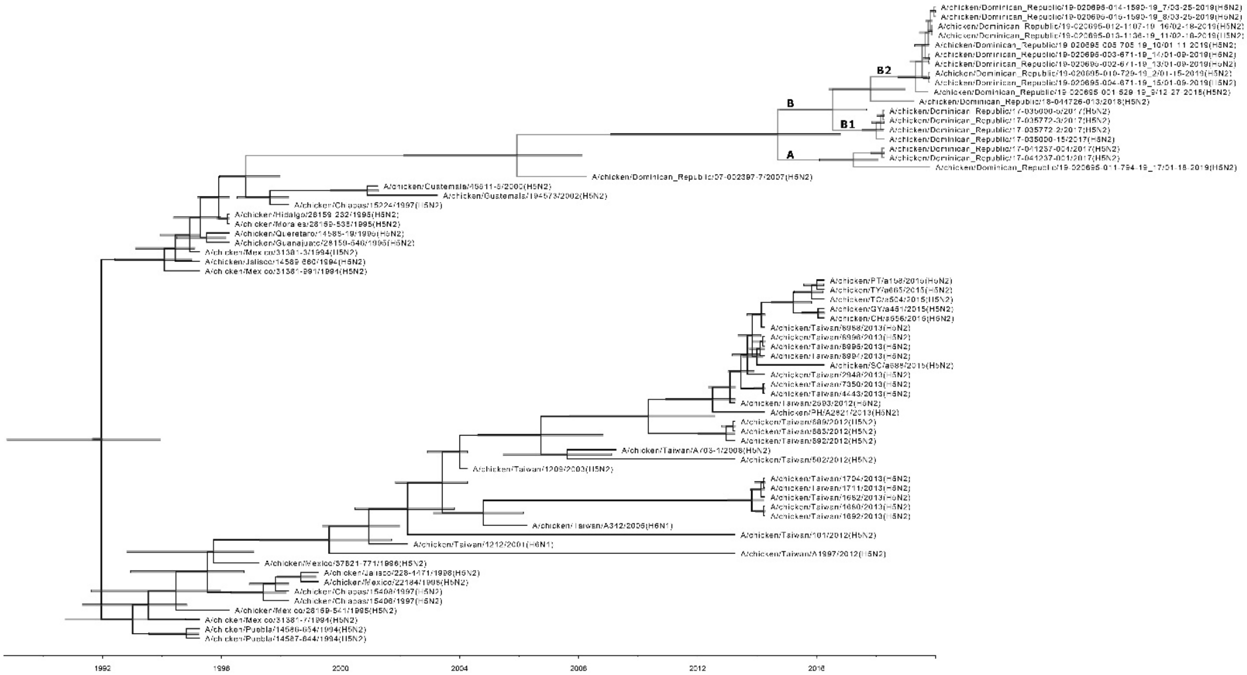

Appendix Figure 5. Maximum-likelihood phylogeny for the NP genome segment sequences of low pathogenicity avian influenza H5N2 viruses, Dominican Republic. Bootstrap values >70% are shown. The scale bar indicates the number of nucleotide substitutions per site.

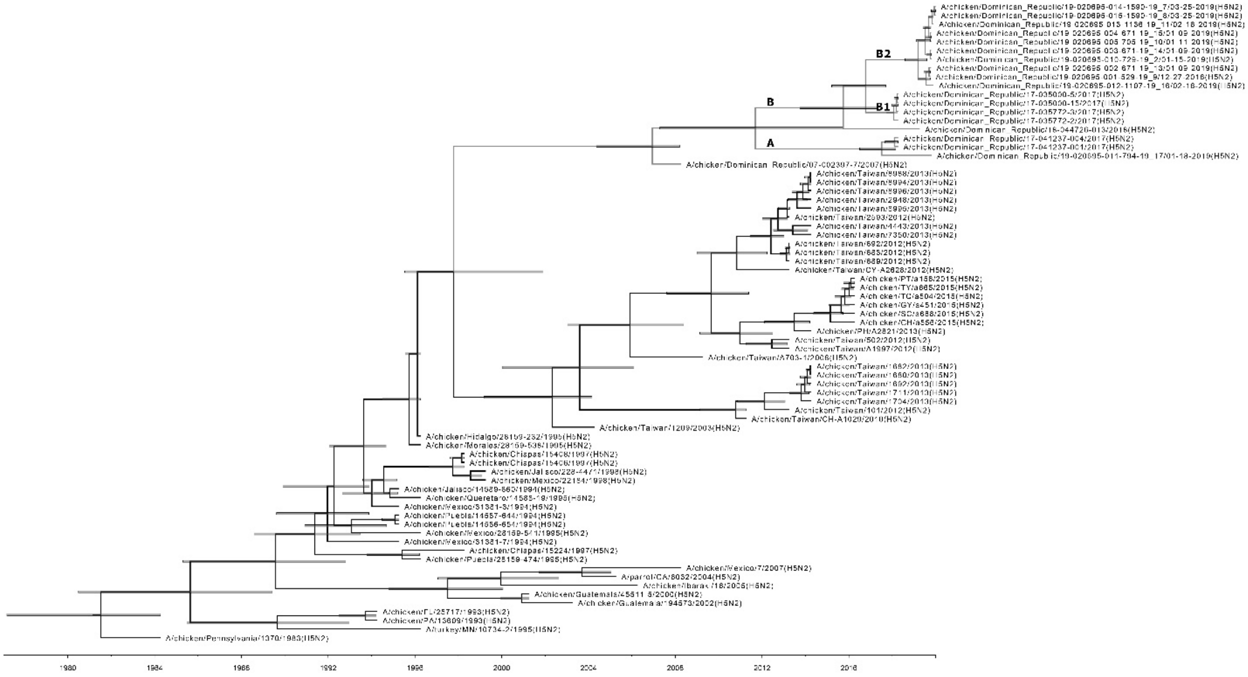

Appendix Figure 6. Maximum-likelihood phylogeny for the NA genome segment sequences of low pathogenicity avian influenza H5N2 viruses, Dominican Republic. Bootstrap values >70% are shown. The scale bar indicates the number of nucleotide substitutions per site.

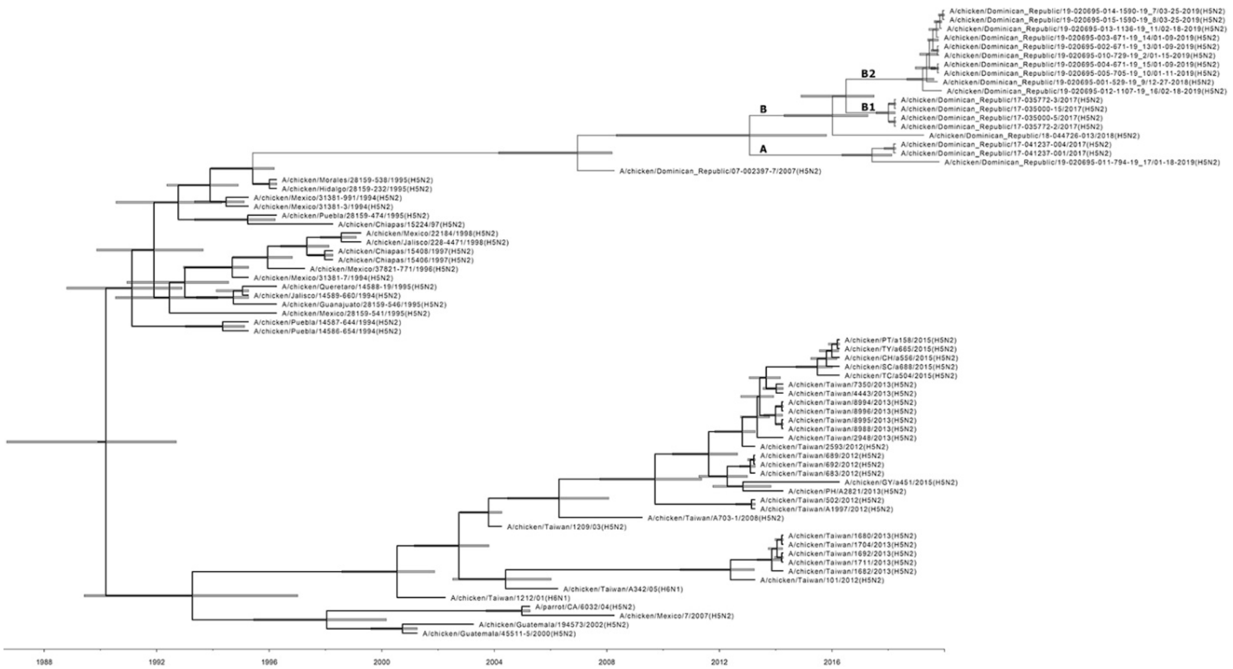

Appendix Figure 7. Maximum-likelihood phylogeny for the M genome segment sequences of low pathogenicity avian influenza H5N2 viruses, Dominican Republic. Bootstrap values >70% are shown. The scale bar indicates the number of nucleotide substitutions per site.

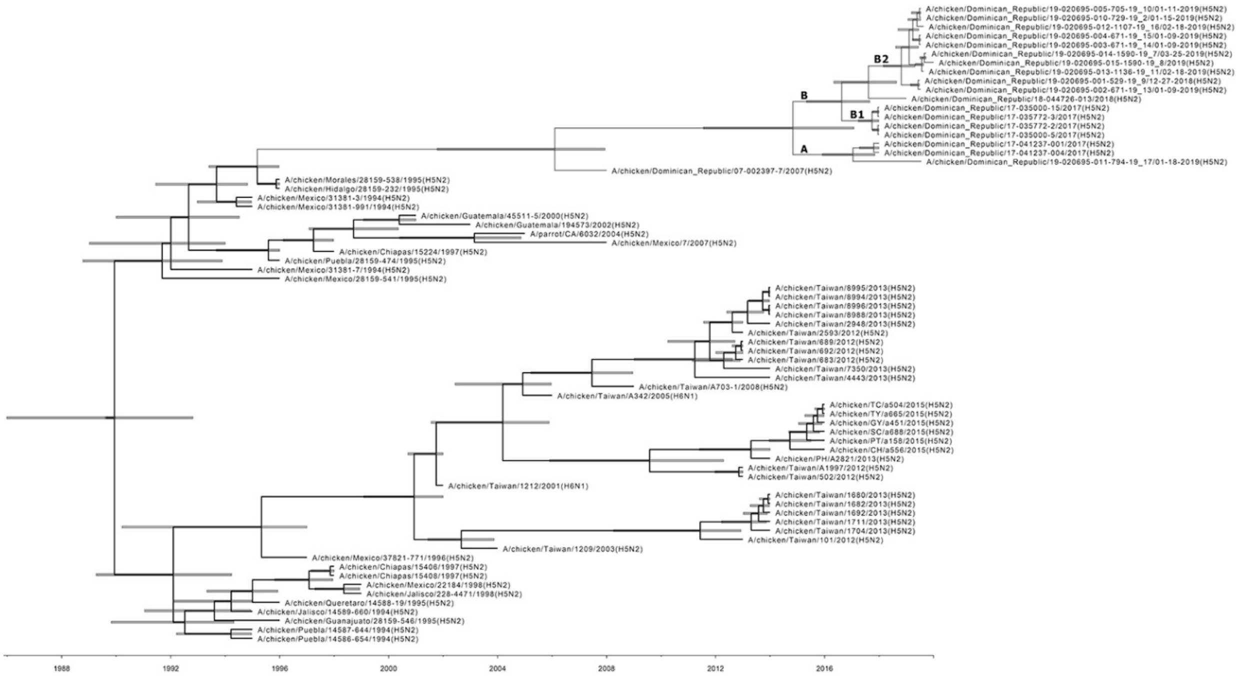

Appendix Figure 8. Maximum-likelihood phylogeny for the NS genome segment sequences of low pathogenicity avian influenza H5N2 viruses, Dominican Republic. Bootstrap values >70% are shown. The scale bar indicates the number of nucleotide substitutions per site.

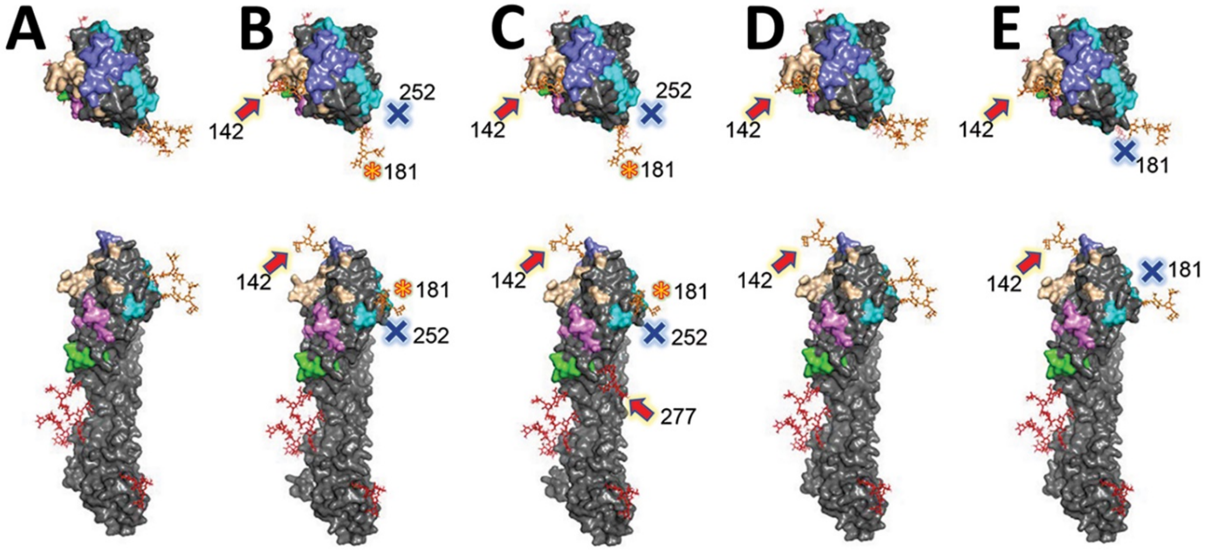

Appendix Figure 9. In silico 3-dimensional (3D) structure prediction and comparison of hemagglutinin protein of low pathogenicity avian influenza H5N2 virus, Dominican Republic. Only monomers are shown. A) CK/DR/07-002397-7/2007; B) genetic cluster A/B/B1 2017–2019; C) genetic cluster B1

CK/DR/035772-3/2017; D) genetic cluster B2, 2018–2019; E) genetic cluster B2 CK/DR/020695-012-1107/2019. Predicted antigenic sites are yellow-orange, slate, green, cyan, and violet. Predicted N-glycosylation sites located on antigenic sites are in orange and the others are represented with red. The N-glycosylation site patterns different from A/CK/Dominican_Republic/07–002397–7/2007 strain are indicated with red arrows for additions, blue crosses for deletions, and orange asterisks for predicted conformational change at antigenic–glycosylation site.
